# Simulation of Solar Cells with Integration of Optical Nanoantennas

**DOI:** 10.3390/nano11112911

**Published:** 2021-10-30

**Authors:** Inês Margarida Pinheiro Caetano, João Paulo N. Torres, Ricardo A. Marques Lameirinhas

**Affiliations:** 1 Department of Electrical and Computer Engineering, Instituto Superior Técnico, 1049-001 Lisbon, Portugal; ines.p.caetano@tecnico.ulisboa.pt; 2 Instituto de Telecomunicações, 1049-001 Lisbon, Portugal; joaotorres@tecnico.ulisboa.pt; 3 Academia Militar, Avenue Conde Castro Guimarães, 2720-113 Amadora, Portugal

**Keywords:** optics, optoelectronic devices, nanoantenna, photovoltaics, plasmonics, semiconductors, solar cells

## Abstract

The evolution of nanotechnology has provided a better understanding of light-matter interaction at a subwavelength scale and has led to the development of new devices that can possibly play an important role in future applications. Nanoantennas are an example of such devices, having gained interest in recent years for their application in the field of photovoltaic technology at visible and infrared wavelengths, due to their ability to capture and confine energy of free-propagating waves. This property results from a unique phenomenon called extraordinary optical transmission (EOT) where, due to resonant behavior, light passing through subwavelength apertures in a metal film can be transmitted in greater orders of magnitude than that predicted by classical theories. During this study, 2D and 3D models featuring a metallic nanoantenna array with subwavelength holes coupled to a photovoltaic cell are simulated using a Finite Element Tool. These models present with slight variations between them, such as the position of the nanoantenna within the structure, the holes’ geometry and the type of cell, in order to verify how its optical response is affected. The results demonstrate that the coupling of nanoantennas to solar cells can be advantageous and improve the capture and absorption of radiation. It is concluded that aperture nanoantennas may concentrate radiation, meaning that is possible to tune the electric field peak and adjust absorption on the main layers. This may be important because it might be possible to adjust solar cell performance to the global regions’ solar spectrum by only adjusting the nanoantenna parameters.

## 1. Introduction

Energy demand has been steadily increasing since the Industrial Revolution and should continue to increase, accompanied by global population growth [1,2,3,4,5]. Although renewable energy use is increasing globally, as of 2018 these sources of energy only accounted for less than 20% of the world’s primary energy consumption [5]. Fossil fuels such as oil, coal and natural gas still meet much of the world’s energy demands [3,4,5]. Concerns over climate change and the energy demand of a rapidly growing global population require an urgent shift towards renewable resources for energy production. Renewables are forecast to become the leading source of primary energy consumption by 2050, with wind and solar representing 70% of total renewable generation [3,4,5].

Nowadays, photovoltaic (PV) cells can directly convert the power associated with solar radiation into DC electric power at a relatively low cost (in comparison with other renewable energies, such as hydro or wind) [3,4]. In addition, the fact that PV cells are robust structures that require very little maintenance makes them an extremely attractive technology, which is rapidly expanding throughout the global energy market [6]. However, PV solar cells have some limitations. Only a small fraction of the incoming solar radiation can be converted to electricity by semiconductors due to the materials’ characteristics and electrical properties.

Even though many research efforts have been put into place to improve the efficiency rates of PV solar panels, the optimization of these efficiency rates has yet to be achieved. Most panels present efficiency rates of around 20% or less, usually requiring a mechanical sun-tracking system to optimize the conversion. Nanotechnology can be part of the solution to this problem [7]. The nanotechnology and optical materials fields have experienced a rapid development, leading to the emergence and development of new systems and devices such as the nanoantenna. With the implementation of this technology, PV solar devices are expected to become more efficient and low-cost [8].

In this research paper, the connection between nanotechnology and photovoltaics is going to be studied through numerical simulation of a 2D and 3D nanoantenna structure coupled to the solar cells. The optical response of such structures is going to be discussed and compared. Finally, based on that analysis, the main goal is to understand if nanoantennas integration into solar cell systems can actually bring advantages or additional improvements to the solar energy harvesting process and, among of all the models simulated, which one is the best suited for such a purpose. Nanoantenna integration into solar cells could eventually constitute a breakthrough in solar energy harvesting on PV panels, due to the solar irradiance concentration and amplification resulting in more incident energy and, therefore, more output power generation. If so, it could be possible to obtain different absorption spectra using the same materials.

### 1.1. Nanoantennas for Solar Energy Harvesting

Photovoltaic panels are currently the most used technology for converting solar energy into electricity. Although PV technology has improved a lot in recent years, it is still not able to fully utilize the abundance of solar energy reaching the earth in the visible and infrared regions and the reradiated infrared energy [9]. Moreover, as a quantum device, the efficiency of PV is a function of the bandgap and the match of the bandgap to the solar spectrum [10]. Furthermore, PV materials are typically only operational during daylight hours and require direct solar radiation for optimal efficiency.

Optical antennas are devices that convert propagating optical radiation into localized energy and vice versa; as a periodic array [11] they could act as a frequency-selective surface (FSS) [11]. They can control and manipulate optical radiation at subwavelength scales [11]. Thus, if coupled with PV solar panels, their overall system efficiency is expected to be higher than current solar panel efficiency. Optical nanoantennas are similar to microwave and radiofrequency (RF) antennas and can couple electromagnetic radiation in the visible and infrared, just like other antennas do at their corresponding wavelengths [9]. Although similar, there are crucial differences between optical antennas and their microwave and RF counterparts due to their nano size and the resonant metal nanostructures’ properties [12].

#### 1.1.1. Theory of Operation

Electromagnetic waves induce time-varying electric fields in metals that apply a force on the electron gas of electrons inside the material, causing the charge carriers to oscillate in a collective movement [13]. This phenomenon is known as surface plasmon and it constitutes the basis for the optical phenomena that gives rise to the unique optical properties associated with nanoantennas, being extremely valuable for the harvesting and absorption of solar energy.

If the resonant frequency of the designed nanoantenna matches the incoming wave length frequency, it leads to the absorption of the incoming electromagnetic (EM) radiation [13]. The efficiency in the absorption of the incident radiation depends on the antenna design and the impedance matching of the antenna. Nevertheless, modelling and experimental measures performed in [9] show that nanoantennas can eventually absorb close to 90% of the available energy [13]. Furthermore, nanoantennas can utilize both the visible and the near IR regions of the spectrum light, as well as the reradiated near IR energy, capturing the sun’s energy during the day but also the radiation of the Earth at night [1,9,14,15,16,17,18,19,20].

#### 1.1.2. Technical Framework

During recent years, a lot of research has been developed regarding different configurations and properties of nanoantennas for various applications. However, these devices still face some challenges and there is a need for improvements. Some of the challenges associated with nanoantennas concern the physical creation of the device, since its dimensions are of the order of nanometers—which is 10^−6^ times smaller than typical RF antennas. Additionally, the device must be capable of efficiently capturing all the incident radiation polarization [21,22,23]. To do this successfully without the need for a sun tracking system means that the acceptance angle must always be as wide as possible. It is also important to consider the impedance and capacitance of the antenna. Furthermore, in order to create an efficient nanoantenna, the choice of materials and design of the device are crucial.

In this study, the analysis will be focused on the implementation of a metallic aperture nanoantenna. More specifically, a metallic subwavelength aperture nanoantenna array. Aperture nanoantennas are able to increase coupling efficiency of nearby light by focusing it onto an aperture region which enables light confinement and better control of the radiation pattern [24]. Since the practical use of subwavelength apertures to enhance light–matter interactions first took place, the interest in the extraordinary optical transmission (EOT) phenomenon has continued to increase, and has already led to ample research on the origin of the phenomenon, the influence of several design parameters (aperture shape and dimensions, metal permittivity, metal adhesion layer) and the development of practical applications type of aperture. Arranging the apertures in a subwavelength array with a periodic pattern, it can provide extra coupling capabilities. However, there are dependencies regarding hole size, lattice spacing, metal film thickness and angular dispersion. Several other parameters also have significant importance such as type of metal, symmetry in the dielectric–metal–dielectric layer stack, finite-size effects of the lattice and the hole shape [15,25]. The transmission peaks observed in the far-field and the intensity enhancement in the near-field, characteristic of the aperture array, are consequent of two different resonant phenomena: the resonant excitation of surface plasmon waves at the metal–dielectric interface and the localized plasmon modes on properly shaped apertures [25].

## 2. Theoretical Foundations

Classic diffraction theories do not predict the existence of extraordinary optical transmission (EOT), nor the generation and propagation of surface plasmon polaritons (SPPs), which give rise to the unique optical properties that nanoantennas are associated with. Hence, these phenomena cannot be analyzed or supported by such theories. Wave propagation theories regarding light–matter interactions can better describe the overall phenomenon.

### 2.1. Light–Matter Interactions

#### 2.1.1. Surface Plasmon

A plasmon is defined as a quantum of a plasma oscillation. In the same way that light consists of photons, plasmon oscillation consists of plasmons. Thus, plasmons are collective oscillations of the electrons in a plasma. These oscillations are traveling waves with a well-defined frequency and wave vector; when such oscillations exist at the interface between a conductor and a dielectric, such as a dielectric–metal interface, they are called surface plasmons (SPs). Plasmon oscillations can couple light in the form of a surface wave, creating surface plasmon polaritons (SPPs). A polariton is the result of strong coupling between electromagnetic waves and an electric or magnetic dipole [16,17,18]. For a dipole structure, such as an array of slits, an aperture or even a corrugation on the surface, upon which light is incident, the photons can excite coherent fluctuations of free electron charges at the metal boundary, creating plasmon oscillations. Such oscillations can couple with the incident light, creating polaritons that propagate at the interface [16,17,18].

The resonant interaction between the surface charge oscillation and the electromagnetic field of the light gives rise to unique properties. The presence of SPPs helps to concentrate and channel light using subwavelength structures, which leads to an electric field enhancement that can be used to manipulate interactions between light and matter and improve the transmission of light by the structure [19,26]. However, for polaritons to be created, the metal needs to exhibit an electric permittivity with a negative real part at the frequency of the incident light. Hence gold, silver, platinum, or aluminum are typically the materials of choice, since all of them satisfy this condition. Additionally, the vector component of the incident light parallel to the interface should be matched with the wave number of the SPP [19].

#### 2.1.2. Extraordinary Optical Transmission

The Bethe–Bouwkamp theory predicted that the power transmitted through the slit would decrease as the diameter of the slit decreased far below the radiation wavelength. However, these predictions were refuted in 1998 when Ebbesen observed the so-called extraordinary optical transmission (EOT) phenomenon for the first time [19]. Extraordinary optical transmission (EOT) is defined as an optical phenomenon in which a structure containing subwavelength apertures in an opaque screen transmits more light than would be expected. In EOT, the nanostructure enables a several orders of magnitude larger transmission efficiency than that predicted by the classical aperture theory [22]. In Ebbesen experiments, this nanostructure was an array of cylindrical holes, with a certain periodicity constant, hole diameter and thickness. The array displayed sharp peaks in transmission for wavelengths as large as ten times the hole’s diameter. For these wavelengths, the transmission efficiency (normalized to the total holes area) could even exceed unity. These unique optical properties are due to plasmons coupling with light on the metal surface, thus creating polaritons (SPPs) [27]. The EOT phenomenon offers the possibility of a variety of applications due to the high transmission efficiencies and the high local field enhancements that can be achieved [15].

## 3. Model Structure and Configuration

To analyze the problem, 2D and 3D simulations of different solar cell structures were computed. All these simulations were developed using a finite element tool, which simulates a physics-based systems described by partial differential equations (PDEs) [1,2,17,18]. All the simulations use FEM to solve Maxwell’s equations in the frequency domain, with the total electrical field being the dependent variable governed by the following equation:

(1) $$\nabla \times \left(\mu_{r}^{-1}\nabla \times E\right)-k_{0}^{2}\times \varepsilon_{r_{c}}\times E=0$$

where *µ_r_* is the relative permeability, *e_rc_* is the relative permittivity, *k*_0_ is the wave number, and **E** is the electric field. In 2D models, the electric field varies with the out-of-plane wave number *k_z_* as presented in Equation (2):

**E**(*x*, *y*, *z*) = **E**_e_(*x*, *y*)*e*^−^*^ikzz^* (2)

### 3.1. 2D Model

**Figure 1:**
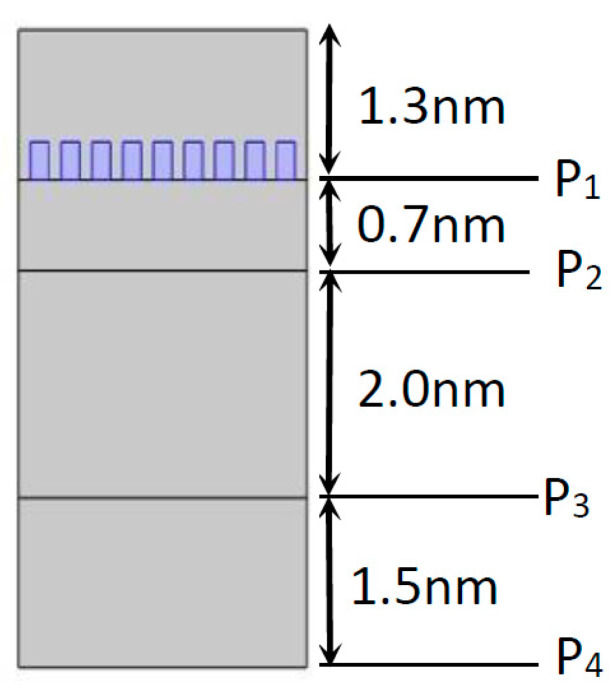
Layout of the 2D a-Si solar cell, with the thickness of each layer and the exact location of each probe.

The 2D model geometry simulated is represented in Figure 1 and is constituted by an amorphous silicon (a-Si) cell and by a dielectric layer on top, which in this case is air. Although a typical solar cell has n-type and p-type layers, the three layers represented in the nanostructure are all composed of intrinsic amorphous silicon, since at room temperature all the impurities of n- and p-type materials are ionized, and from an optic point of view, these two materials act similarly to the behavior of the intrinsic material. The light absorption in a solar cell occurs mainly in the intrinsic region of the cell; hence, it can be assumed that the n-type and p-type doped layers do not contribute to the generation of photocurrent in the solar cell [1,2,20]. Therefore, for the purpose of simplifying the following simulations, the nanostructure illustrated in Figure 1 is considered as an actual a-Si solar cell. An aperture aluminum (Al) nanoantenna was introduced on top of the silicon cell, as illustrated in Figure 1, to study the differences in the optical response of the cell with and without a nanoantenna.

In frequency domain analyzes it is necessary to characterize the optical constants *n* and *k* of each material, where *n* is the refractive index and *k* the extinction coefficient. The models used for each material are retrieved from an online database [28]. In both versions of the 2D model, the incident electric field, defined by |*E*_0_| = 1 µV/m, is generated on top of the structure. The electric field amplitude should be parallel to the horizontal nanoantenna array in order to reach the nanoantenna’s apertures in a tangential manner. For a metal nanoantenna in the visible and near-infrared regions, the EM field wave vector must have a parallel component to the metal surface in order for polaritons to be originated and, therefore, for the EOT phenomenon to take place [1,16,17,18]. Hence, the incident electric field amplitude is defined in the *x* axis direction, as |*E_x_*| = |*E*_0_| V/m and |*E_y_*| = |*E_z_*| = 0 V/m. The EM field amplitude varies in the x direction, but the EM field wave propagates in the *y* axis direction.

### 3.2. 3D Model

#### 3.2.1. Amorphous Silicon (a-Si) Solar Cell Structure

**Figure 2:**
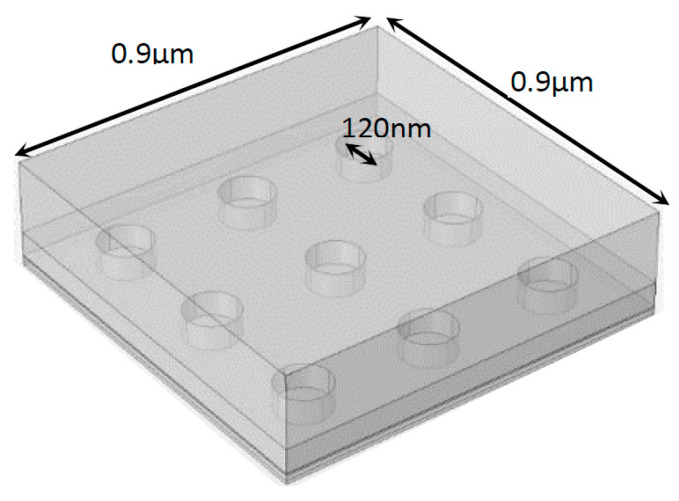
Layout of the 3D a-Si solar cell with the inclusion of an aluminum nanoantenna.

To further deepen the study of a solar cell coupled with a nanoantenna it is important to analyze the performance of the 3D models. The environment and conditions defined in a 3D model resemble actual reality in a more realistic way, so the optical response given by the cell and the occurrence of the EOT phenomenon can be examined in a more detailed and accurate way. Similar to what was established in 2D, two versions of the 3D model were created: without and with a nanoantenna (Figure 2). The materials of the 3D model were the same as those implemented in the 2D model. An electric field, defined by |*E*_0_| = 1 µV/m, was generated on the top of the 3D model external dielectric layer boundary. The EM field was perpendicular to the nanoantenna, having the opposite direction of the *z* axis, and it was defined in the generation port by |*E_x_*| = |*E_y_*| = (*E_o_/*$\sqrt{2}$) V/m and |*E_z_*| = 0 V/m. The remaining simulation requirements and specifications were equivalent to the ones applied in both versions of the 2D model, as previously described. Additionally, those requirements and specifications were the same for both 3D models, with and without the nanoantenna.

#### 3.2.2. CIGS Solar Cell Structure

**Figure 3:**
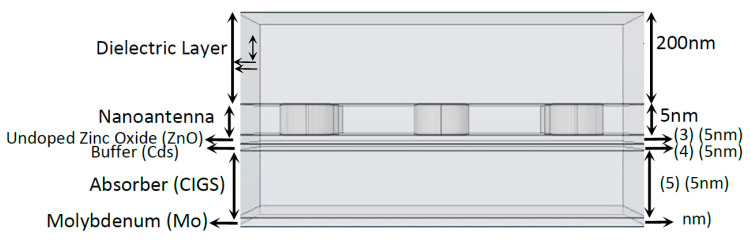
Layout side view of the 3D CIGS solar cell structure with the composition and thickness of each layer.

The study and assessment of the output optical response of a 3D model using a different types of solar cell would be noteworthy [2]. Therefore, based on the original 3D model, a 3D model with a CIGS (Copper Indium Gallium Selenide) solar cell structure was developed, as shown in Figure 3. The specifications and conditions that were imposed on this model and corresponding simulation were the same as those defined for the previous models.

## 4. Simulation Results

The simulations that follow were accomplished using four different boundary probes, one at the end of each layer of the structure. Those probes analyzed the maximum ratio between the electric field norm and the incident electric field norm, |*E*/*E*_0_|. If the simulated structure had the ability to transmit more light than its incidence, then the value obtained for the ratio was greater than 1, indicating the occurrence of extraordinary optical transmission.

### 4.1. 2D Simulations

**Figure 4:**
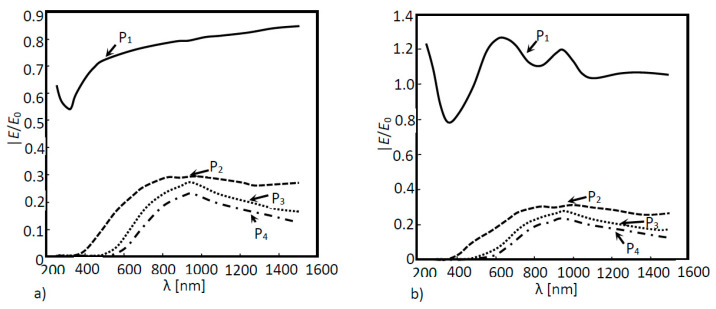
Normalized electric field as a function of the wavelength for a 2D a-Si solar cell structure. Measures were performed in four different positions: P_1_, P_2_, P_3_ and P_4_ for two different situations: (**a**) without a nanoantenna and (**b**) with a nanoantenna.

In Figure 4a, 2D a-Si simulation was performed for two situations: (a) without a nanoantenna and (b) with the inclusion of a nanoantenna. As seen in Figure 4a, the maximum ratio did not reach values above or even equal to 1, meaning that the EOT phenomenon did not take place. However, this result was expected, since without the presence of any subwavelength aperture, SPPs will not be generated, and extraordinary transmission cannot happen. It is important to underline that SPPs constitute the main agents of the EOT phenomenon in metallic nanoantennas functioning in the visible and near-infrared region of the light spectrum. Finally, since the electric field amplification is not verified in any layer, the electric field will simply decay along the propagation distance. When analyzing Figure 4b, it is possible to verify that the EOT phenomenon did in fact occur, since values of |*E*/*E*_0_| greater than 1 were obtained for boundary probe 1. This will be the boundary that presents the best results, since it was located directly underneath the nanoantenna array, which is where surface plasmon polaritons were generated and propagated. However, the EOT phenomenon occurrence did not influence the results of the remaining boundary probes. In fact, the values obtained by these probes were quite similar to those obtained without the nanoantenna. In order for the results of those probes that monitored the second and third layer of the a-Si cell to be almost unaltered, the majority of the absorption had to have occurred in the first layer of the cell.

The above results show the electric field that was not absorbed in the solar cells. These values should be as low as possible. It is crucial to establish that, the greater the difference between the input electric field in the cell or in a certain layer and its output electric field, the greater the absorption is. Thus, one can assume that the greater the gap between the normalized electric field values from one probe to the next, the greater the absorption is, bound by those probes. In other words, more photons are absorbed in the layer and, consequently, more electric power can be generated.

**Figure 5:**
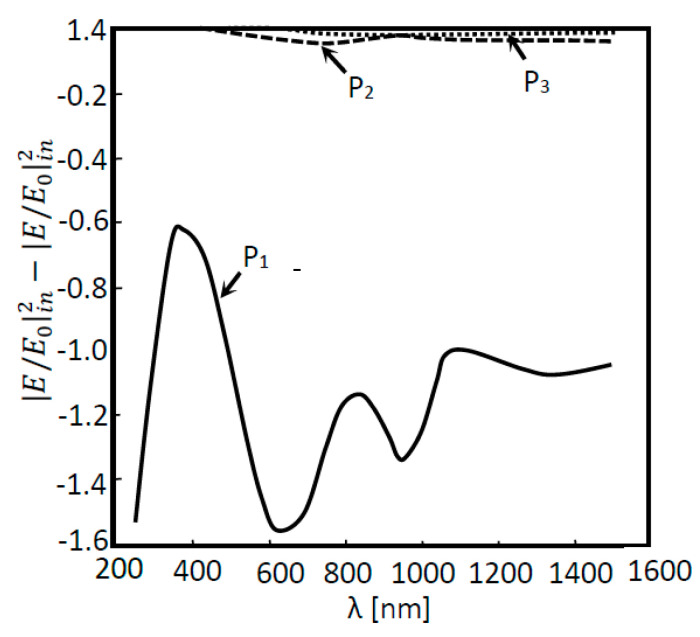
Absorption as a function of the wavelength for the 2D a-Si solar cell structure with the inclusion of a nanoantenna.

In Figure 5, the absorption is shown as a function of the wavelength for the several layers in the solar cells. In Figure 5, the absorption in each layer of the cell is also shown. Thus, for layers where absorption occurs, these values will be less than zero. Therefore, according to Figure 5, photons are essentially absorbed in the first layer of the cell (Layer 1), particularly for the incident wavelength regions where field amplification is shown (Figure 4b), such as for the wavelengths of 600 nm and 1000 nm. These results corroborate the conclusions found by the analysis of Figure 4b. Taking into account the fact that, due to field amplification, the nanoantenna inclusion implies that more electrons were available in the first layer of the cell, and since the majority of the absorption takes place in this layer, then more electrons will be absorbed, resulting in higher electric current generation.

### 4.2. 3D Simulations

The procedures and conditions applied to carry out the 3D simulations, with and without the inclusion of a nanoantenna into the cell, are identical to those considered for the 2D model simulations.

#### 4.2.1. Amorphous Silicon (a-Si) Solar Cell Structure

**Figure 6:**
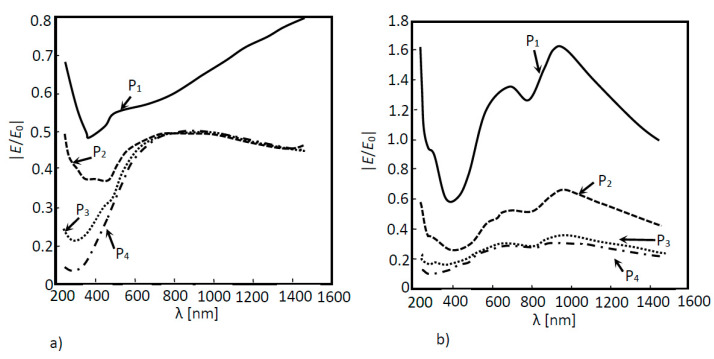
Normalized electric field as a function of the wavelength for a 3D a-Si solar cell structure. Measures were performed in four different positions: P_1_, P_2_, P_3_ and P_4_ for two different situations: (**a**) without a nanoantenna and (**b**) with a nanoantenna.

As expected, for the amorphous silicon solar cell model without the nanoantenna (Figure 4a), the simulation results in Figure 6a show that EOT did not occur. This situation was already observed in the 2D models; due to losses suffered throughout the air layer and the absorption suffered throughout the a-Si cell, the electric field decays along the structure. On the other hand, the results in Figure 6b clearly showed the presence of EOT, though only for boundary probe 1, located below the nanoantenna array, at the beginning of the solar cell—possibly due to the reduced propagation distance of the SPPs (around nm). This boundary also shows two peaks in the normalized electric field values, which were not present in the optical response of the model without the nanoantenna. These peaks were obtained for the incident wavelengths of 700 nm and 1000 nm, located in the visible and near-infrared regions, respectively. However, the extraordinary transmission verified in boundary probe 1 did not affect the remaining boundary probes, as the obtained electric field ratio values were quite small, never reaching unitary values.

**Figure 7:**
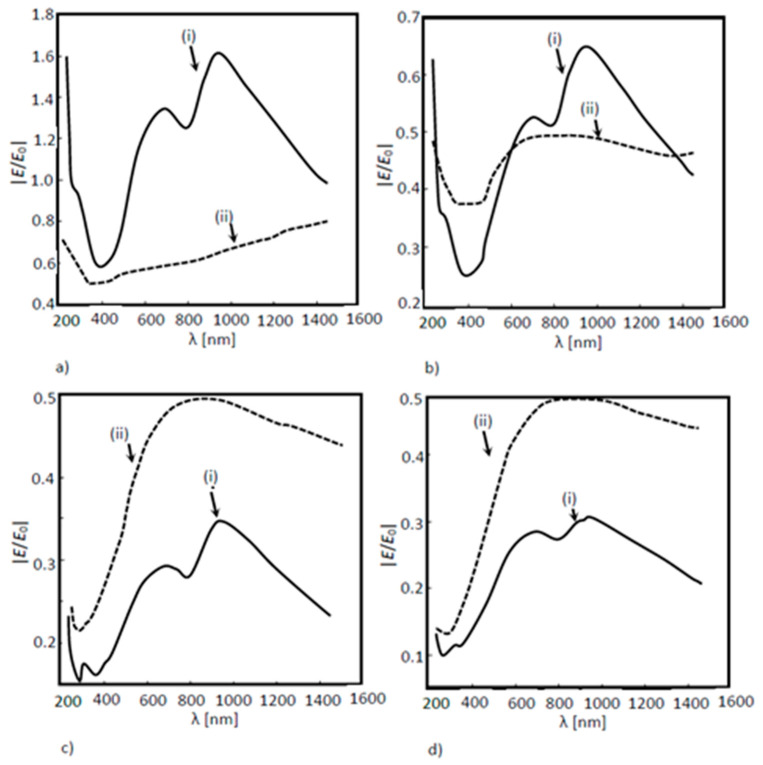
Normalized electric field as a function of the wavelength for a 3D a-Si solar cell structure. Measures were performed in four different positions: (**a**) P_1_, (**b**) P_2_, (**c**) P_3_ and (**d**) P_4_ for two different situations: (i) without a nanoantenna and (ii) with a nanoantenna.

In Figure 7a, it was possible to see the difference in the electric field values between the two structures; with and without the inclusion of a nanoantenna. In this last situation, EOT was observed all over the wavelength range, from the visible to the near infrared region. On the other hand, Figure 7b shows that, although EOT was not verified, there was still a field amplification for the wavelength range between 600 nm and 1400 nm. Figure 7c,d, shows a higher field absorption along the solar cell in the model with the nanoantenna inclusion, when compared with the results acquired for the model without the nanoantenna included.

**Figure 8:**
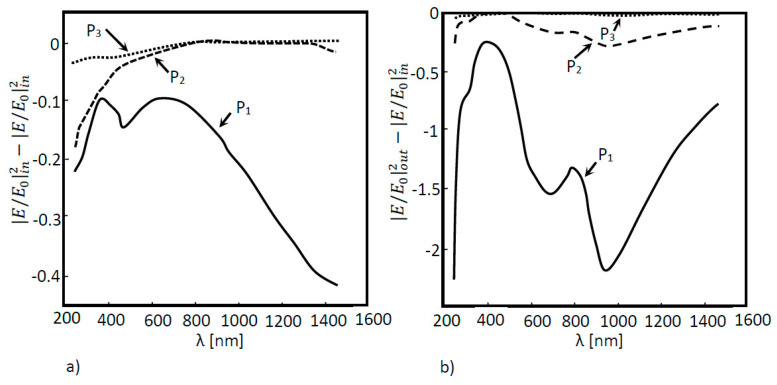
Absorption as a function of the wavelength for the simulated 3D a-Si solar cell structure. Measures were performed in three different positions: P_1_, P_2_ and P_3_ for two different situations: (**a**) without a nanoantenna and (**b**) with a nanoantenna.

This conclusion is supported by Figure 8a,b, where absorption curves for both situations (with and without nanoantennas) are shown. By analyzing these figures, it becomes clear that for the model with the nanoantenna, the absorption was higher. The absorption increases was noticed in all the layers of the solar cell, but was especially accentuated in the first layer, where EOT occurred. Moreover, absorption peaks were directly related to the field amplification peaks observed in Figure 6b.

#### 4.2.2. Amorphous Silicon (a-Si) Solar Cell Structure: Nanoantenna Inclusion within the Solar Cell

**Figure 9:**
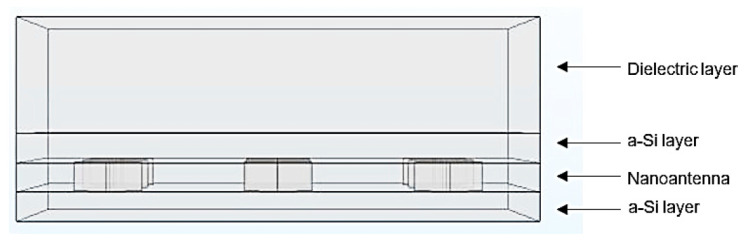
Side view of the 3D a-Si solar cell structure with the nanoantenna inclusion within the solar cell.

The nanoantenna location inside the nanostructure may be an important factor when it is concerned to the system optical response. Due to this, a study regarding the nanoantenna inclusion in the solar cell, between the a-Si layers, was carried out. It is important to mention that in order to include the nanoantenna into the structure, the solar cell dimensions had to be changed, which means that the system optical responses cannot be directly compared. The model in question is shown in Figure 9.

**Figure 10:**
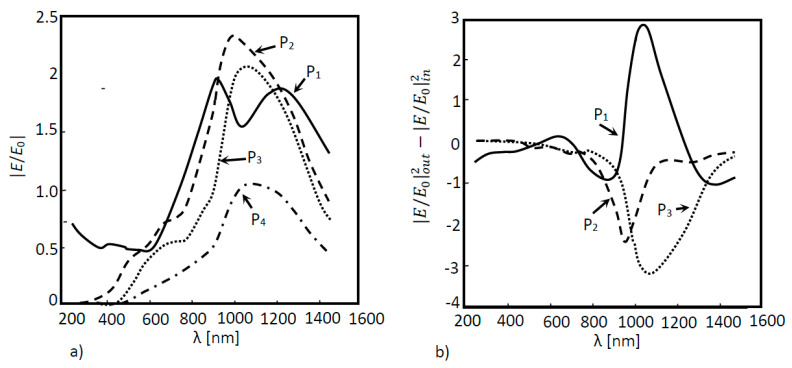
(**a**) Optical response of the simulated 3D a-Si solar cell structure as a function of the wavelength with a nanoantenna within the solar cell and (**b**) Absorption curves of the simulated 3D a-Si solar cell structure as a function of the wavelength.

In Figure 10a, the optical response simulation is shown as a function of the wavelength of the above structure for each layer (P_1_, P_2_, P_3_ and P_4_), and in Figure 10b, the absorption for the same layers and structure is shown as a function of the wavelength. According to Figure 10, the extraordinary optical transmission was evident at boundaries P_1_, P_2_ and P_3_. Although the nanoantenna was located between probes 2 and 3, probe 1 was also influenced by the EOT phenomenon. It was also noticed that this phenomenon was confined to the near infrared region. Due to the nanoantenna location, the incident light that reached the nanoantenna had already passes through one layer of a-Si (the first layer of the cell), meaning that photons with energy equal to or higher than the Si energy bandgap (1.6 eV corresponding to 775 nm), had already been absorbed. This means that only the light in the wavelength higher than 775 nm would pass thought the nanoantenna. Figure 10b supports this conclusion, where a substantial absorption increase in the second and third layers (layer 2 and layer 3) was noticed in the near infrared region of the spectrum.

#### 4.2.3. Amorphous Silicon (a-Si) Solar Cell Structure: Inclusion of a Nanoantenna with Conical Holes

**Figure 11:**
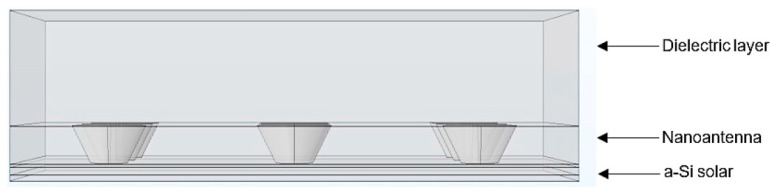
Side view of the 3D a-Si solar cell structure with nanoantenna with conical holes.

The nanoantenna hole’s geometry is also an important parameter when analyzing the output optical response of a solar cell. In order to analyzed the influence of this feature, conical holes (see Figure 11) were considered and optical response as a function of the wavelength was studied.

**Figure 12:**
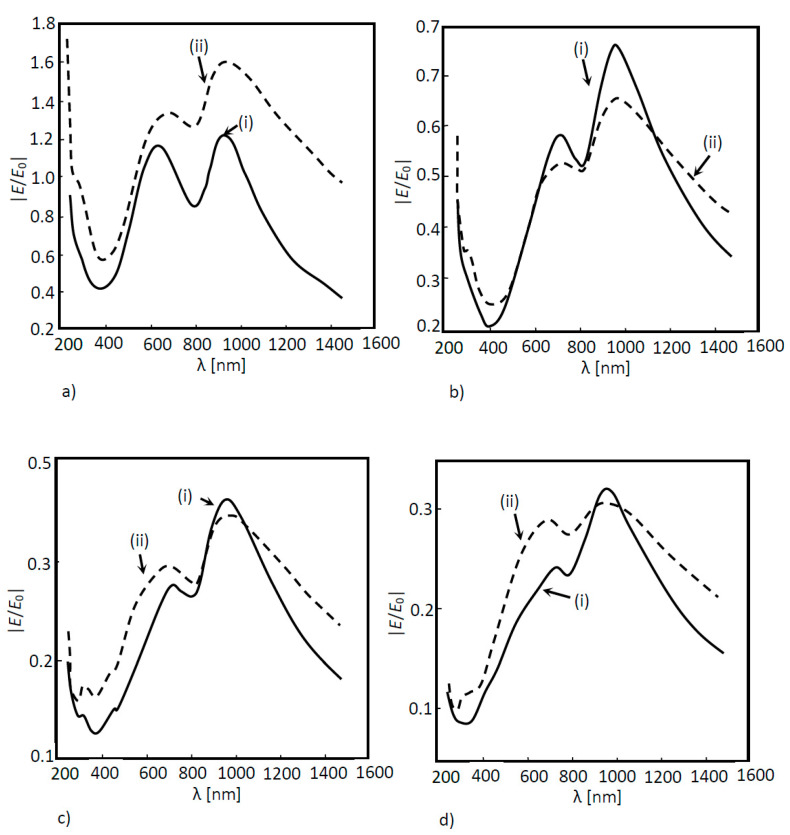
Normalized electric field as a function of the wavelength for a 3D a-Si solar cell structure for two different configurations of nanoantenna hole apertures: (i) conical structures; (ii) cylindrical structures. The normalized electric field was measured between layers as shown in (**a**) probe 1; (**b**) probe 2; (**c**) probe 3 and (**d**) probe 4.

In Figure 12, the results of the solar cell optical response with nanoantennas with cylindrical holes and conical holes are compared. Since the nanoantenna hole geometry may eventually influence the occurrence of the EOT phenomenon, the results at boundary probe 1 are particularly relevant, since this boundary is located directly beneath the nanoantenna, where EOT takes place. According to Figure 12a, nanoantennas with conical holes, instead of cylindrical ones, did not result in a higher extraordinary transmission, since for the model with cylindrical holes, the results clearly showed a greater field amplification in boundary probe 1, and in the remaining boundary probes, both models produced similar output optical spectra.

#### 4.2.4. CIGS Solar Cell Structure

**Figure 13:**
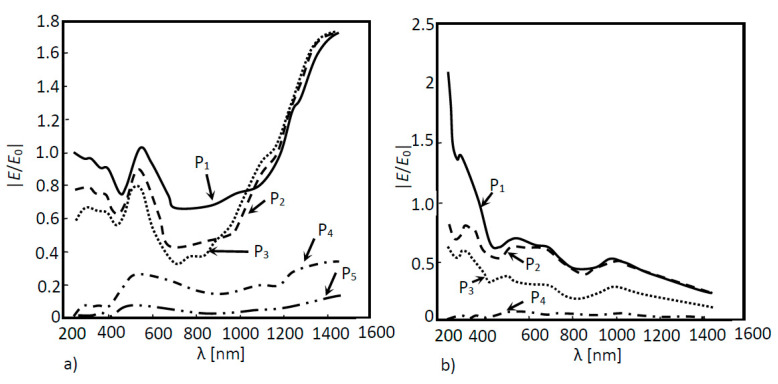
Normalized electric field as a function of the wavelength for a 3D CIGS structure. Measures were performed in four different positions: P_1_, P_2_, P_3,_ P_4_ and P_5_ for two different situations: (**a**) without a nanoantenna and (**b**) with a nanoantenna.

The adopted methodology used to simulate this structure is similar to those defined previously. In the following simulations, the maximum normalized electric field absolute values for five different boundary probes located at the end of each layer were measured. Through the inspection of Figure 13a, it is possible to notice that, for the first three boundary probes, the normalized electric field reached values equal to 1 or higher, even without the nanoantenna in the system—especially in the near infrared spectrum region.

This amplification was directly related to a positive imaginary component of the dielectric function of the two first CIGS solar constituents: ZnO and Cds. However, it is important to note that almost no absorption occurred throughout these first two layers of the cell, as verified in Figure 13a. In fact, the results in this figure also showed that the third layer (*P*_3_) was the only layer where absorption occurred in a significant way. In these solar cells, the absorption typically occurred in the infrared region, since CIGS cells absorb radiation in the infrared region of the spectrum—as their energy band gap can be tuned roughly from 1.01 eV to 1.68 eV, corresponding approximately to 1230 nm and 740 nm, respectively.

**Figure 14:**
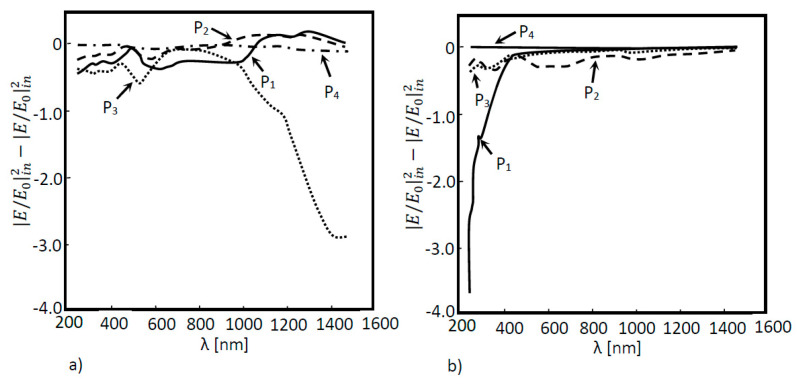
Absorption curves of the simulated 3D CIGS structure: (**a**) without the inclusion of a nanoantenna; (**b**) with the inclusion of a nanoantenna.

With the nanoantenna inclusion on top of the CIGS solar cell, the behavior of the solar cells’ optical response to the wavelength, shown in Figure 13b, was quite different when compared to the case without a nanoantenna. In the near infrared region, the normalized electric field values were quite low for all the probes when compared to those obtained without a nanoantenna. An increase in the normalized electric field was also noticed in boundary probe 1 as the wavelength decreased for values near the UV region, around the wavelength of 250 nm in the UV zone, and the EOT phenomenon was exhibited between the range of 250 nm and 400 nm. According to Figure 14b, the absorption in the CIGS layer (layer 3) was significantly lower in this cell when compared to the case without a nanoantenna. Nevertheless, absorption did increase in the second layer of the cell (Cds layer), and especially in the first layer of the cell (ZnO layer). Additionally, most of the absorption of photons throughout all the layers concerned photons in the UV region of the light spectrum.

## 5. Conclusions

The main goal of this investigation was to study the behavior of a solar cell when coupled with an optical nanoantenna. The coupling of these devices on solar cells could eventually constitute a breakthrough regarding solar energy harvesting on PV panels, due to the concentration and amplification of solar irradiance—resulting in more incident energy and, therefore, more generation of output power. The 2D and 3D simulations of different solar cell structures, with and without a nanoantenna, allowed the observation and assessment of how the optical response of such structures is influenced by the presence of the nanoantenna, and if extraordinary optical transmission (EOT) occurs.

Based on 2D and 3D results regarding the a-Si solar cell structure, it can be confirmed that EOT does always occur when an aluminum nanoantenna is present in the solar cell structure, which ultimately implies that these structures are able to transmit more light than its incidence. The results also proved that this phenomenon was linked to the generation and propagation of SPPs, since high amplification and concentration of the electromagnetic field near the dielectric–metal interfaces was always verified, constituting clear evidence of the presence of surface plasmon polaritons—as expected by [1,16,17,18], and by the same order of values. Additionally, an increase in absorption was noticeable when the nanoantenna was coupled to the cell, especially in the first layer (between boundary probe 1 and boundary probe 2). It is also noteworthy that in both 2D and 3D simulation, the obtained optical responses for the models with nanoantennas showed a peak in the normalized electric field in the visible and infrared regions. These indicate that the implementation of nanoantennas in solar cells could be pertinent for applications using different parts of the light spectrum.

Furthermore, the results suggest that the location of the nanoantenna inside the cell structure affected the light spectrum region where EOT took place. This peculiarity might prove to be useful for certain applications—for example, for converting light into electricity. Regarding the impact of the nanoantenna hole shape in the structure’s optical response, the obtained results produced quite similar spectra to those obtained by the 3D model with a nanoantenna with cylindrical holes—except when it came to the normalized electric field values, mainly in boundary probe 1, located directly beneath the nanoantenna, for which the model with cylindrical holes did present, for the entire wavelength range, higher electric field values. This signals to the fact that EOT is more accentuated in this type of model, therefore leading to the conclusion that the conical hole’s geometry is perhaps unsuitable.

Finally, simulations regarding the CIGS solar cell structure illustrated underwhelming results regarding the occurrence of EOT in this particular model, where evidence of extraordinary transmission was only found in boundary probe 1 for a very small range of the incident wavelength. Nevertheless, the field amplification that did occur was seen in the UV region of the light spectrum, which can prove the nanoantenna’s benefit for certain applications within that range of incident wavelengths. Overall, it was demonstrated that extraordinary optical transmission (EOT) does occur when an aluminum nanoantenna is coupled with a solar cell and, more importantly, that the structure produces promising results regarding field amplification and increases in the absorption of photons throughout the solar cell.

## Data Availability

Not applicable.
